# Clinical pharmacist interventions in elderly patients with mental disorders in primary care focused on psychotropics: a retrospective pre–post observational study

**DOI:** 10.1177/20451253211011007

**Published:** 2021-04-22

**Authors:** Matej Stuhec, Lea Lah

**Affiliations:** Faculty of Pharmacy, University of Ljubljana, Askerceva Cesta 7, Ljubljana, SI-1000, Slovenia; Department of Clinical Pharmacy, Ormoz Psychiatric Hospital, Ormoz, Slovenia & Department for Pharmacology, Maribor, Slovenia & Faculty of Pharmacy Ljubljana, University of Ljubljana; University of Ljubljana, Ljubljana, Slovenia

**Keywords:** clinical pharmacist, long-term interventions, mental disorders, primary care setting, psychotropics

## Abstract

**Background:**

Mental disorders pose a significant clinical burden and affect approximately one-third of older adults. Although studies have shown positive impacts of clinical pharmacist (CP) interventions within the general population, the long-term effects of such cooperation on geropsychiatric patients in primary care settings are not yet known. This study evaluated whether CP interventions have a long-term impact on the quality of medication prescribing in geropsychiatric patients.

**Methods:**

We conducted a retrospective non-interventional observational pre–post study for the 2015–2017 period, involving patients aged 65 or above for whom a medication review was provided by a CP. The study included participants with mental disorders treated with polypharmacy, including at least one psychotropic. Potentially inappropriate medications (PIMs) in elderly patients were determined with the Priscus list, and potential type X drug–drug interactions (pXDDIs) with Lexicomp®. Up-to-date treatment guidelines were used to evaluate patient pharmacotherapy, and patient medication was evaluated before the initial medication review and again 6 months later.

**Results:**

The study included 48 patients (79.4 years, SD = 8.13) receiving a total of 558 medications (155 for the treatment of mental disorders). The number of medications decreased by 9.5% after the medication review. The CP proposed 198 interventions related to psychotropics, of which 108 (55%) were accepted by the general practitioners. All accepted (99.1%) interventions except one were still maintained 6 months after the interventions had been proposed. They led to a significant decrease in the total number of medications, PIMs, and pXDDIs (*p* < 0.05), and improved treatment guidelines adherence.

**Conclusions:**

CP interventions decreased the number of medications, PIMs, and pXDDIs, and almost all interventions were maintained 6 months later. These results provide evidence for the positive effects of CP interventions in a primary care setting. Additional research with a larger sample size and a randomized study design is needed.

## Introduction

Mental disorders impose a significant clinical and economic burden globally and affect about one-third of older adults. A cross-sectional study of 35 general practices in Ireland estimated the prevalence of mental health disorders in older adults to be 19.1%. The prevalence was associated positively with age and increased from 14.8% in the 55–59 age group to 28.9% in the 80–84 age group. The most common mental disorders were depression (17.1%), panic/anxiety (11.3%), cognitive disorders (5.6%), and alcohol (3.8%) and substance misuse (3.8%).^1^ Mental disorders in this population are most often treated with psychotropics, due primarily to difficulties with other interventions and a lack of resources (e.g., for psychotherapy). Although effective, psychotropics are often over-prescribed in this population, and more prudent prescribing strategies are needed to minimize the risks of over-prescribing. Psychotropics, especially antidepressants, anxiolytics, and antipsychotics, have several important adverse effects and drug–drug interactions (DDIs), which can lead to treatment failure and serious harm. Potentially inappropriate medications (PIMs) in elderly patients can be avoided using several PIM lists (e.g., Priscus, STOPP/START, Beers), which also list many psychotropics.^2–4^ PIMs, in particular antipsychotics and anxiolytics, are commonly used in older adults. An Austrian study of most nursing homes in Voralberg (*n* = 1844) found that 70.3% of all residents had at least one PIM 70.3% [95% confidence interval (CI) 67.2–73.4] and that 1014 (55%) residents were using at least one psychotropic PIM. The most frequently prescribed PIMs were prothipendyl (25.9% of residents), lorazepam (14%), and diclofenac (6.1%). A multiple regression analysis showed an inverse association of PIMs with cognitive impairment and significant positive associations with permanent restlessness and permanent negative attitude.^5^ Furthermore, many geropsychiatric patients are treated with irrational polypharmacy: unnecessary polypharmacy used while there are alternative treatments with fewer medications that are safer and/or more effective.^6,7^ Both PIMs and irrational polypharmacy can result in harm, treatment failure, and increased treatment costs.^8,9^ In Slovenia, most patients with mental disorders are treated in primary care, which accounts for two-thirds of antidepressants and the majority of anxiolytics prescriptions, so further research on psychotropics in primary care is needed.^10^

One approach to minimizing PIMs and irrational polypharmacy is the inclusion of clinical pharmacists (CPs) in primary care settings where general practitioners (GPs) work, which has been the subject of research, particularly in the United States (US).^11^ Despite some recent research examining large populations, there are no data on the long-term acceptance of CP recommendations in primary care in Central Europe.^6,7^

This study evaluates the long-term impact of CP interventions in a primary care setting on the quality of medication prescription, as measured by the number of overall medications, PIMs, and DDIs in geropsychiatric patients treated with polypharmacy. We hypothesized a negative association between clinical pharmacist interventions and the number of PIMs, medications, and potential type X drug-drug interactions (pXDDIs, contraindicated potential DDIs as defined by Lexicomp®) as well a positive association between CP interventions and treatment guidelines adherence.

## Methods

### Study design and inclusion/exclusion criteria

We conducted a non-interventional retrospective observational pre–post study. It included patients serviced by the Ljutomer primary health center in southeast Slovenia who had been referred to a CP between 1 February 2015 and 1 July 2017. Patients were selected according to GP medical referral forms (no impact on selection criteria), which are used by GPs in Slovenia to refer patients to various medical specialists. The CP performed medication reviews for patients with a medical referral from their GP. A medication review with recommendations was sent back to the respective GP, who accepted or rejected the recommendations at the patient’s next visit. This service is paid for by the Health Insurance Institute of Slovenia. Each patient was considered a separate observational episode. For each patient, the data were collected at their first visit to their GP after the CP’s medication review and again 6 months later (long-term acceptance).

The study included patients aged 65 years or above at the time of their initial examination, who were concurrently treated with five or more medications (i.e., polypharmacy), including at least one psychotropic (ATC code N Nervous system including psychotropics) and were diagnosed with at least one mental disorder as defined by the 10th revision of the International Statistical Classification of Diseases and Related Health Problems (ICD-10).^12^ Only patients without missing data were included. The STROBE Statement checklist was used to insure the inclusion of all items required in reports of observational studies.^13^ The patients’ health data, including diagnoses, were obtained from their medical documentation (patient charts and the CP medication reviews).

The CP in this study was an experienced CP (PharmD/PhD) with over 10 years of work experience in a psychiatric hospital (ward rounds, medication reviews, meetings with patients and psychiatrists). The CP did not communicate with the GPs and patients after providing a medication review. The GPs also did not provide feedback on why they accepted or rejected the CP’s recommendations. The CP did not have any conversation with GP and patients after the visit, and, therefore, pharmacotherapy after 6 months was also checked (long-term acceptance).

### Interventions and data collection

The selection criteria were applied to all patients referred to the CP by GPs. The CP provided a medication review, which was given to the GPs who made the final decision on whether to accept or reject the proposed changes. The Slovenian Pharmacy Act allows the pharmacist to conduct medication reviews in primary care settings and hospitals. In primary care, all GPs can refer patients to CPs. In hospitals, CPs are part of the multidisciplinary teams on the wards and provide medication reviews for inpatients.^14^ For details on the medication review service, see our other studies and reports.^7,14^ Medication changes were retrieved from patients’ medical charts. This study examined only three main intervention types: individual drug discontinuation (ineffective medication, no indication), drug initiation (untreated indication, re-initiation), and drug dosage adjustment (dose change, dose frequency, titration, or renal function adjustment). The results of the CPs’ interventions were determined by examining the patients’ medical records after the medication review was produced. This study only included prescription medication, so over-the-counter medications, dermal preparations, and medications on an as-needed basis were excluded.

Researchers (LL and MS) examined all medication reviews and patient charts, classified the changes into intervention types as well as noted if the accepted recommendations were maintained 6 months after the GPs received the medication review (long-term acceptance). All data were retrieved retrospectively, so there was no direct contact between the researchers and GPs or patients. LL was an MPharm student and MS is an experienced psychiatric CP with over 10 years of work experience in a psychiatric hospital, including daily rounds and ward activities, and ambulatory clinical pharmacy service. The latest guidelines for the treatment of individual diseases were considered for this study. The PRISCUS list was used to determine PIMs in the elderly.^2^ Only pXDDIs as defined by Lexicomp® 3.0.2, were included in the study, as in our previous study.^15^ The impact on treatment guidelines adherence was also evaluated with various treatment guidelines.

### Outcomes

The primary outcomes (number of medications, PIMs, pXDDIs) were noted after the medication review and 6 months later. The long-term acceptance was calculated as the difference between 6 months and immediate GP acceptance). In addition, the authors examined several treatment guidelines (secondary outcome) to evaluate the impact of CP recommendations on treatment guidelines adherence.^2–4,16–18,19–20^ For this purpose, all patients with all various diagnoses (e.g., schizophrenia, insomnia, dementia) were included and guidelines adherence was assessed case by case. When treatment guidelines provided insufficient data, various studies and summaries of product characteristics were used to determine if the medication use was appropriate.

### Statistical analysis

The main characteristics of the sample were described using descriptive statistics. Patient medical records were retrieved immediately after the first conversation with the CP when the medication review was performed and 6 months later. The Shapiro–Wilk test was used to test for data normality. A *t* test for the dependent samples (paired *t* test) was used for normally distributed variables, and a non-parametric Wilcoxon signed-rank test was used for non-normally distributed variables. Patients with missing data were excluded from the study, which also addressed loss to follow up. Bias was assessed as descriptive bias (see Discussion). The *p* value used was 0.05 and the required sample size was not calculated. The analyses were performed with the Statistical Package for Social Science 22.0 for Windows® (SPSS, Chicago, IL, USA).

## Results

### General results

**Figure 1.**
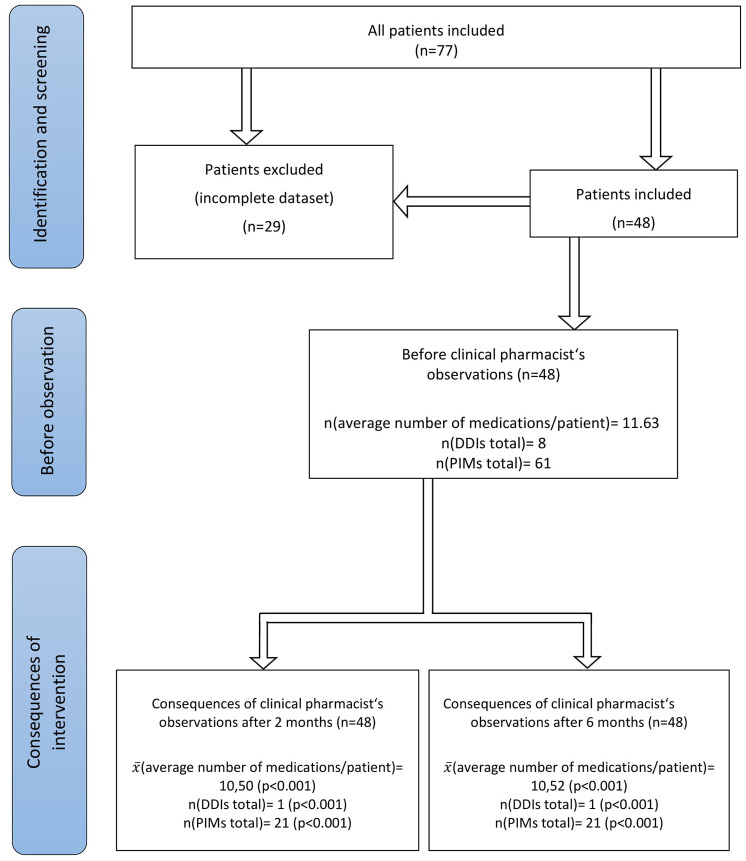
A flow chart of the main study outcomes. DDI, drug–drug interaction; PIM, potentially inappropriate medication.

The sample included 48 patients [79.4 years, standard deviation (SD) = 8.13] (Figure 1, Flowchart). The mean number of diagnoses per patient was 4.96 (median = 5). Dementia (50%), schizophrenia (29%), and depression (19%) were the most prevalent. In total, the participants were treated with 558 different medications (mean = 12.6 medications, median = 11), of which 155 (28%) were psychotropics and used to treat mental disorders (3.2 psychotropics per patient); 38 patients (79.2%) were treated with at least one antipsychotic and 30 patients (62.5%) with an antidepressant.

### Primary outcomes

The CP proposed 198 interventions (134 drug discontinuations, 45 drug initiations, and 19 drug adjustments), which amounted to 4.1 interventions per patient (median = 4), of which the GPs accepted 108 (55%). The mean of accepted interventions per patient was 2.25 (median = 2) (*p* < 0.05). The highest number of accepted interventions was eight (in one patient). In 36 patients (75%), the GPs accepted all proposed recommendations. The interventions were maintained 6 months after introduction in all patients.

**Figure 2.**
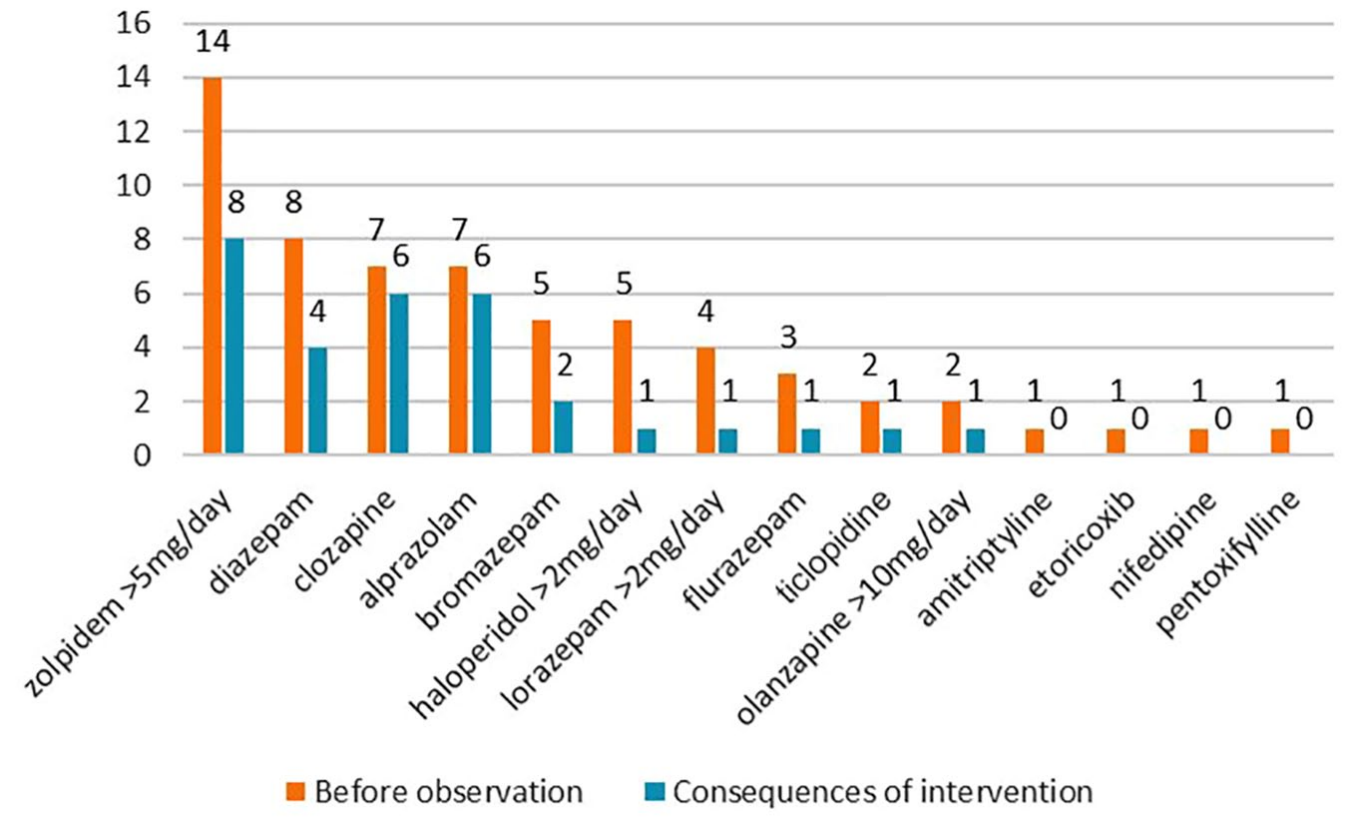
Number of patients with PIMs before medication review and after 6 months according to the PRISCUS list.

Before the medication review, 61 PIMs (as defined by the PRISCUS list) were prescribed, which represented 10.3% of all prescribed medications. On average, patients received 1.05 PIMs (median = 1). Detailed results are presented in Figure 2. After GPs received and accepted or rejected the proposals in the medication review, the total number of PIMs decreased significantly from 61 to 31, which is a 49% reduction (*p* < 0.05). The average number of PIMs per patient also decreased from 1.05 to 0.7 (post-review median = 1). Psychotropics represented 91.8% of all PIMs and 39.3% of all PIMs were benzodiazepines. The most common PIM was the hypnotic zolpidem (23% PIMs) followed by diazepam (13% PIMs). The number of pXDDIs also decreased significantly after the interventions from 8 to 1 (*p* < 0.05).

### Secondary outcomes

Patients were grouped according to their diagnoses and medications. Table 1 shows the full review of treatment groups according to the proportion of accepted interventions and treatment guidelines adherence. In the three largest groups of patients, the CP interventions improved treatment guidelines adherence (*p* < 0.05).

**Table 1.** Comparison of treatment guidelines adherence in patient groups before and after the medical review.

Patients group	Depression	Anxiety	Insomnia	Dementia	Schizophrenia
No of diagnosis	30	26	24	19	38
No of proposed interventions	19	11	12	3	15
No of accepted interventions	11	5	4	3	9
Treatment guidelines adherence (before) % patients	33.3% (*n* = 10)	61.5% (*n* = 16)	29.2% (*n* = 7)	89.4% (*n* = 17)	71.1% (*n* = 27)
Treatment guidelines adherence (after) % patients	73.3% (*n* = 22)	80.8% (*n* = 21)	54.2% (*n* = 13)	100.0% (*n* = 19)	89.5% (*n* = 34)
Difference	+40.0% (*p* < 0.05)	+19.3% (*p* < 0.05)	+25.0% (*p* < 0.05)	+11.6% (*p* = 0.157)	+18.4% (*p* < 0.05)

## Discussion

This study is the first retrospective pre–post study assessing the impact of a CPs’ medication review service in geropsychiatric patients in Central Europe. Our results provide three key findings and suggest the service may be beneficial in a primary care setting.

Firstly, the results suggest that the proposed medication changes were relatively well accepted, as over 90% of the recommendations were still maintained 6 months after introduction, although the patients’ clinical outcomes were not measured. Acceptance rates may vary between different work environments, as the rate in this study was higher than in our previous study of a nursing home, but lower than in a study of a Slovenian psychiatric hospital (88.0%).^6,15,21^ A CP in a psychiatric hospital is in daily contact with psychiatrists and patients (e.g., during ward rounds), whereas primary care patients do no receive follow up with a CP, unless referred by their GP anew. Future research could evaluate the effects of additional appointments with CPs and the effects of independent prescribing by CPs, which is currently not possible in Slovenia, but is elsewhere (e.g., collaborative plan agreements in the US).^11^

Secondly, CP recommendations may curb irrational polypharmacy. In this study, the service reduced the total number of medications, which corroborates our previous studies in a primary community setting and a nursing home.^6,15^ This service can thus strongly reduce polypharmacy and associated negative effects in line with the goals of health insurance bodies, such as the Health Insurance Institute of Slovenia (Slovene: Zavod za zdravstveno zavarovanje Slovenije – a funding body in Slovenia), which funds the service described in this paper.^15^ We found psychotropics were often involved with several PIMs and pXDDIs, which we also observed in our previous studies.^6,15,21^ Additionally, nearly all pXDDIs were removed by the medication review service, which is a larger reduction than in our previous studies.^6,15^ The remaining pXDDI (between rivastigmine and propranolol), was due to the patient needing both beta-blockers for heart failure and rivastigmine for dementia. Other pXDDIs were combinations containing quetiapine (three times: metoclopramide, trospium and amiodarone), olanzapine (once: trospium), clozapine (once: carbamazepine) and rivastigmine (twice: metoclopramide, propranolol). Our results show that antipsychotics were often part of pXDDIs and that the CP removed all important pXDDIs involving antipsychotics and antidepressants, which is a larger reduction than in our previous studies.^6,15,21^ Furthermore, the total number of PIMs (PRISCUS list) also decreased. The largest PIM reduction (32%) occurred with hypnotics and sedatives (e.g., zolpidem, bromazepam, alprazolam, and diazepam) (Figure 1), which is in line with recommendations,^2–4^ and reflects our previous studies, as benzodiazepines should be avoided in elderly patients due to their negative effect on falls and cognitive decline.^5–7^

The third finding is that the service improved treatment guidelines adherence. Most commonly, the interventions were related to depression treatment. Improved depression treatment adherence was also reported in a US study, where this service is available in some states.^11^ However, the CP in our study did not have prescribing rights, which could be explored in future studies. A study with a large sample size reported that depression is not adequately recognized and treated in primary care: although 51.6% (95% CI, 46.1–57.2) of 12-month cases received treatment for depression (*n* = 9,090), the treatment was adequate in only 41.9% of them (95% CI, 35.9–47.9), resulting in 21.7% (95% CI, 18.1–25.2) of 12-month depression cases being treated adequately.^22^ The most commonly proposed interventions regarding antidepressants in our study were sertraline initiation (often instead of escilatopram, which featured in pXDDIs), and mirtazapine and trazodone initiations (often instead of benzodiazepines). This is also in line with the guidelines.^18^ Insomnia guidelines adherence improved, as benzodiazepines were often discontinued in line with the guidelines and the PRISCUS and Beers lists.^2,3^ For schizophrenia treatment, discontinuation of small doses of quetiapine was recommended in some cases, as such use is common in clinical practice despite poor evidence for it.^18^ Our results reflect the findings of our 2019 study examining antipsychotic treatment guidelines, in which 9 out of 21 different CP interventions (42.8%) were accepted by GPs. The acceptance rate of the recommendations, but not patient age, improved treatment guidelines adherence for antipsychotics (*p* = 0.041) and quetiapine was found to be the most frequently used antipsychotic, prescribed to 30 out of 49 patients included in the study (61.2%).^7,10^ Some important dose adjustment interventions were also provided in dementia treatment.

Despite positive results, this study has several important limitations. The researchers did not contact the study participants directly to gather data on their response to the service. The methodology used in this study has limitations that may affect the results and introduce high bias (i.e., no control group, no randomization, selection bias, no outcomes measuring, polypharmacy, heterogeneous population, small sample size, monocentric study, risk of type II error). These could all be addressed in future studies. Despite these limitations, we provide valuable data on the effects of CP interventions in a primary care facility in Slovenia. The study adds to the research on healthcare provision for elderly patients with mental disorders in Central Europe and demonstrates what effects can be expected from implementing the service in countries that do not yet have it.

## Conclusion

This study shows that CP recommendations in geropsychiatric patients treated with polypharmacy improved treatment guidelines adherence and the quality of pharmacotherapy prescribing by reducing the total number of medications, pXDDI, and PIMs. The accepted interventions remained mostly unchanged 6 months after their introduction. Additional research with a larger sample size or a prospective study design would be needed to attempt to replicate these results.

## References

[1] McCombe G Fogarty F Swan D, et al. Identified mental disorders in older adults in primary care: a cross-sectional database study. Eur J Gen Pract 2018; 24: 84–91. 29353511 10.1080/13814788.2017.1402884 PMC5795746

[2] Holt S Schmiedl S Thürmann PA. Potentially inappropriate medications in the elderly: the PRISCUS list. Dtsch Arztebl Int 2010; 107: 543–551. 20827352 10.3238/arztebl.2010.0543 PMC2933536

[3] Gallagher PF Barry PJ Ryan C, et al. Inappropriate prescribing in an acutely ill population of elderly patients as determined by beers’ criteria. Age Ageing 2008; 37: 96–101. 17933759 10.1093/ageing/afm116

[4] O’Mahony D Gallagher P Ryan C, et al. STOPP & START criteria: a new approach to detecting potentially inappropriate prescribing in old age. Eur Geriatr Med 2010; 7: 45–51.

[5] Mann E Haastert B Böhmdorfer B, et al. Prevalence and associations of potentially inappropriate prescriptions in Austrian nursing home residents: secondary analysis of a cross-sectional study. Wien Klin Wochenschr 2013; 125: 180–188. 23536016 10.1007/s00508-013-0342-2

[6] Stuhec M Bratović N Mrhar A. Impact of clinical pharmacist’s interventions on pharmacotherapy management in elderly patients on polypharmacy with mental health problems including quality of life: a prospective non-randomized study. Sci Rep 2019; 9: 16856. 31728029 10.1038/s41598-019-53057-w PMC6856189

[7] Stuhec M Gorenc K. Positive impact of clinical pharmacist interventions on antipsychotic use in patients on excessive polypharmacy evidenced in a retrospective cohort study. Global Psychiatry 2019; 2: 155–164.

[8] Lau DT Kasper JD Potter DEB, et al. Hospitalization and death associated with potentially inappropriate medication prescriptions among elderly nursing home residents. Arch Intern Med 2005; 165: 68–74. 15642877 10.1001/archinte.165.1.68

[9] lbert SM Colombi A Hanlon J. Potentially inappropriate medications and risk of hospitalization in retirees: analysis of a US retiree health claims database. Drugs Aging 2010; 27: 407–415. 20450238 10.2165/11315990-000000000-00000 PMC2929124

[10] Slovenian Health Insurance Institute Prescription Database (report for 2018), https://www.nijz.si/sl/publikacije/poraba-ambulantno-predpisanih-zdravil-v-sloveniji-v-letu-2018 (accessed 20 April 2020).

[11] Finley PR Bluml BM Bunting BA, et al. Clinical and economic outcomes of a pilot project examining pharmacist-focused collaborative care treatment for depression. J Am Pharm Assoc 2011; 51: 40–49. 10.1331/JAPhA.2011.09147 21247825

[12] 10th revision of the International Statistical Classification of Diseases and Related Health Problems (ICD), a medical classification list by the World Health Organization (WHO), https://www.who.int/classifications/icd/en/ (accessed 9 August 2020).

[13] von Elm E Altman DG Egger M, et al. The Strengthening the Reporting of Observational Studies in Epidemiology (STROBE) statement: guidelines for reporting observational studies. J Clin Epidemiol 2008; 61: 344–349. 18313558 10.1016/j.jclinepi.2007.11.008

[14] Slovenian Pharmacy Act, 2016, Official Journal Republic of Slovenia, number 85/16, http://www.pisrs.si/Pis.web/pregledPredpisa?id=ZAKO7375 (accessed 9 April 2020).

[15] Stuhec M Gorenc K Zelko E. Evaluation of a collaborative care approach between general practitioners and clinical pharmacists in primary care community settings in elderly patients on polypharmacy in Slovenia: a cohort retrospective study reveals positive evidence for implementation. BMC Health Serv Res 2019; 19: 118. 30760276 10.1186/s12913-019-3942-3 PMC6375190

[16] Hasan A Falkai P Wobrock T, et al. World Federation of Societies of Biological Psychiatry (WFSBP) Task Force on Treatment Guidelines for Schizophrenia. Guidelines for Biological Treatment of Schizophrenia, part 1: update 2012 on the acute treatment of schizophrenia and the management of treatment resistance. World Federation of Societies of Biological Psychiatry (WFSBP). World J Biol Psychiatry 2012; 13: 318–378. 22834451 10.3109/15622975.2012.696143

[17] Stahl SM. Emerging guidelines for the use of antipsychotic polypharmacy. Rev Psiquiatr Salud Ment 2013; 6: 97–100. 23485567 10.1016/j.rpsm.2013.01.001

[18] Wilson SJ. British Association for Psychopharmacology consensus statement on evidence-based treatment of insomnia, parasomnias and circadian rhythm disorders. J Psychopharmacol 2010; 24: 1577–1601. 20813762 10.1177/0269881110379307

[19] American Psychiatric Association. Practice guideline for the treatment of patients with major depressive disorder. 3rd ed. Arlington, VA: APA, 2010.

[20] Subramanyam AA Kedare J Singh OP, et al. Clinical practice guidelines for geriatric anxiety disorders. Indian J Psychiatry 2018; 60: 371–382. 10.4103/0019-5545.224476 PMC5840911 29535471

[21] Stuhec M. Pharmacotherapy review as a safety and cost tool in patients management in Slovenian psychiatric hospital. V: abstracts of the 27th ECNP congress, Berlin, Germany, 18-21 October 2014. Eur Neuropsychopharmacol 2014; 24: S735–S736.

[22] Kessler RC Berglund P Demler O, et al. The epidemiology of major depressive disorder: results from the National Comorbidity Survey Replication (NCS-R). JAMA 2003; 289: 3095–3105. 12813115 10.1001/jama.289.23.3095

